# Seed Priming with Silicon as a Potential to Increase Salt Stress Tolerance in *Lathyrus odoratus*

**DOI:** 10.3390/plants10102140

**Published:** 2021-10-09

**Authors:** Rasha S. El-Serafy, Abdel-Nasser A. El-Sheshtawy, Amira K.G. Atteya, Abdulrahman Al-Hashimi, Arshad Mehmood Abbasi, Ibrahim Al-Ashkar

**Affiliations:** 1 Horticulture Department, Faculty of Agriculture, Tanta University, Tanta 31527, Egypt; rasha.elserafi@agr.tanta.edu.eg; 2 Environment and Bio-Agriculture Department, Faculty of Agriculture, Al-Azhar University, Cairo 11651, Egypt; 3 Horticulture Department, Faculty of Agriculture, Damanhour University, Damanhour 22516, Egypt; amira.khames@agr.dmu.edu.eg; 4 Department of Botany and Microbiology, College of Science, King Saud University, P.O. Box 2455, Riyadh 11451, Saudi Arabia; aalhashimi@ksu.edu.sa; 5 University of Gastronomic Sciences, 12042 Pollenzo, Italy; amabbasi@cuiatd.edu.pk; 6 Department of Environmental Sciences, COMSATS University, Abbottabad Campus, Islamabad 22060, Pakistan; 7 Department of Plant Production, College of Food and Agriculture Sciences, King Saud University, Riyadh 11451, Saudi Arabia

**Keywords:** *Lathyrus* *odoratus*, seed priming, seawater, antioxidant, proline, SiNPs

## Abstract

Water shortage is a major problem limiting the expansion of green areas and landscapes. Using seawater as an alternative source of potable water is not a novel idea, but the issue of salt stress needs to be resolved. Salinity has a negative impact on growth and the aesthetic value of ornamental plants. In order to overcome these challenges, *Lathyrus odoratus* seeds were hydro-primed and halo-primed with silicon (Si) and silicon nanoparticles (SiNPs), and exposed to seawater levels. Seawater markedly reduced seed germination and growth of *Lathyrus* seedlings, but halo-priming was shown to significantly alleviate its negative effects. Broadly, SiNPs increased the germination percentage, reduced photosynthetic pigments and carbohydrates decrease, and enhanced water relations, despite having a negative effect on germination speed. Halo-priming significantly increased the proline content and the activities of certain enzymatic (SOD, APX and CAT) and nonenzymatic (phenolic and flavonoids) compounds, that positively influenced oxidative stress (lower MDA and H_2_O_2_ accumulation), resulting in seedlings with more salt stress tolerance. Halo-priming with Si or SiNPs enhanced the Si and K^+^ contents, and K^+^/Na^+^ ratio, associated with a reduction in Na^+^ accumulation. Generally, halo-priming with Si or SiNPs increased *Lathyrus* seedlings salt stress tolerance, which was confirmed using seawater treatments via improving germination percentage, seedlings growth and activation of the antioxidant machinery, which detoxifies reactive oxygen species (ROS).

## 1. Introduction

Sweet pea *(Lathyrus odoratus*), an annual herb, belongs to the Fabaceae family, and is an important ornamental plant in temperate regions. *Lathyrus* is a climbing plant that reaches up to 2 m in height using tendrils. It is cultivated for its attractive, strongly fragrant, and decorative flowers. It has a range of colors, including pure whites, pinks, oranges, reds, blues, and lavenders. *Lathyrus* is widely used as a bed plant in landscapes and gardens, and is cultivated for the floral industry. As a member of the legume family, these plants are toxic and should not be eaten.

Currently, many countries are attempting to increase the amount of green areas and landscapes in urban environments, due to their vital role in mitigating climate change, and the impact of heat [1,2], air pollution [3], and water pollution [4]. In addition, they have a positive effect in terms of reducing respiratory illness, allergies, and improving public health [3,5,6]. Expanding green areas is often challenging as a result of potable water deficiency for irrigation; thus, seeking an alternative to clean water is crucial. Using seawater as an alternative to potable water in agriculture was first attempted after the Second World War. The major challenge for using seawater to irrigate landscapes is the elevated salinity. Leaf necrosis and burns are common symptoms of foliage injury in plants irrigated with low-quality or saline water [7,8]. Moreover, it causes a reduction in shoot and roots growth. Despite this, landscape plants are highly variable in terms of salinity tolerance, which can depend on various factors including climatic conditions, soil or substrate type, irrigation method, plant species, and/or growth stage [9,10]. The impacts of salinity on the growth and appearance of landscapes have attracted much attention. This is in part due to its negative influence on the aesthetics and ornamental appearance. Much effort has been focused on inducing salt tolerance, and seed priming is among the most promising methods for achieving this.

Seed priming is a pre-sowing technique that leaves the seed partially hydrated. This hydration only allows the pre-germinative physiological and biochemical metabolic processes, without radical protrusion [11]. Seed priming can accelerate and homogenize seed emergence, enhance the growth and vigor of seedlings [12]. Halo-priming and hydro-priming are types of seed priming. Hydro-priming and halo-priming are defined as soaking seeds in water and salt solutions respectively [13]. Seed priming has been successfully demonstrated to elevate the germination percentage and speed, and enhance seedling vigor under normal and stressed environments [14,15]. Hydro and halo-priming applications enhanced plant growth and performance of wheat under salt stress conditions [16]. Silicon (Si) is a beneficial element commonly used in the halo priming technique, both in bulk size (sodium silicate) and nanoparticle size (SiNPs) [17,18]. Silicon application is an eco-friendly strategy to improve plants’ salinity stress response [19]. It boosts plant resistance to salinity and drought stress [20,21], reduces the negative impact of salt stress on chlorophyll content and biomass production [22], boosts adaptive responses, such as phenolic compound production, mineral uptake, and antioxidant activity [23,24]. Seed priming with sodium silicate enhanced germination characteristics and seedling vigor of wheat plants under drought stress [16]. Halo-priming with SiNPs improved seed germination and seedling growth under salinity stress and normal conditions [18,25].

As a result of the importance of sweet pea cultivars as bedding plants in gardens and landscapes, and the scarceness of potable water for landscapes irrigation, the current study aimed to evaluate the effects of seed priming with (Si) and silicon nanoparticle (SiNPs) treatments on plant growth, leaf water status, and the biochemical and physiological traits in *Lathyrus odoratus* under seawater treatments.

## 2. Results

### 2.1. Germination Characteristics

**Figure 1:**
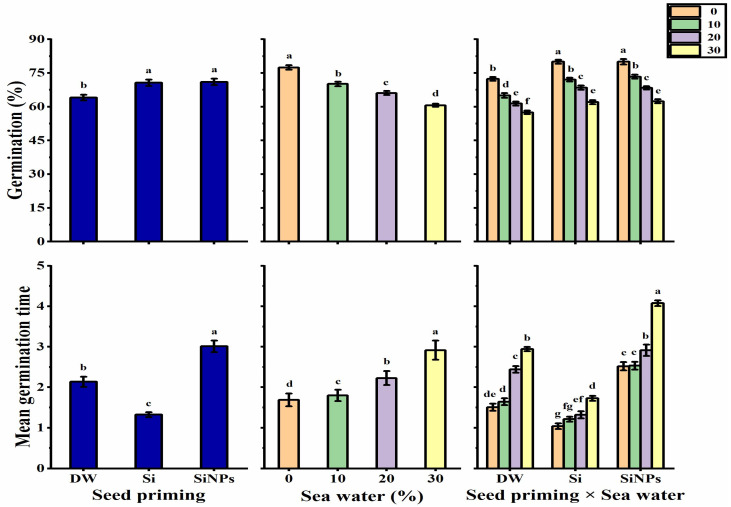
Effect of seed priming on germination (%) and mean germination time of *Lathyrus odoratus* seeds irrigated with seawater levels (0, 10, 20, and 30%). Data are mean value ± SE. Bars with different letters are significantly different at *p* ≤ 0.05 level.

The results in Figure 1 present the effect of seed priming application on germination characteristics of *Lathyrus* seeds exposed to seawater treatments. Halo-priming application with Si and SiNPs significantly improved the germination percentage (GP) of *Lathyrus* seeds as compared with hydro-priming application. Concerning seawater levels, a gradual decrease was observed in the GP with increasing seawater levels, as the highest salinity level (30%) significantly reduced GP by 21.72%, as compared with unsalted treatments (0%). The highest GP values of 80.02 and 80% were recorded with the treatments Si and SiNPS-primed seeds under non-stressed condition, respectively. Meanwhile, the lowest GP value was obtained by hydro-primed seeds under 30% saline condition (57.48%).

Halo-primed seeds with Si had the lowest mean germination time (MGT), which produced 56.03 and 38.32% less than SiNPs and DW, respectively. A significant and growing increase in the MGT value was observed with increased seawater levels, since the highest salinity level (30%) increased MGT by 42.12%, as compared with unsalted treatment (0%). In terms of seed priming and seawater interaction, the lowest MGT was given by Si-primed seeds under non-stressed condition (1.04), while the highest MGT was obtained by SiNPS-primed seeds under 30% saline condition (4.08). 

### 2.2. Plant Growth

**Figure 2:**
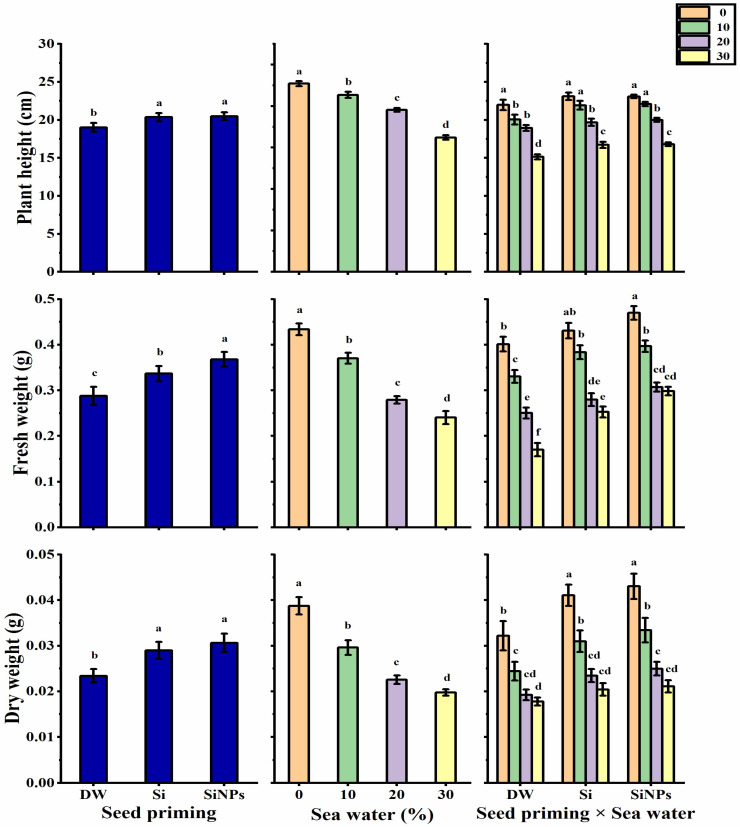
Effect of seed priming on plant height (cm), fresh weight (g), and dry weight (g) of *Lathyrus odoratus* seedlings irrigated with seawater levels (0, 10, 20, and 30%). Data are mean value ± SE. Bars with different letters are significantly different at *p* ≤ 0.05 level.

The height of *Lathyrus* seedlings significantly increased following halo-priming application (Si or SiNPs) as compared with hydro-priming application (Figure 2). Seawater treatments negatively impacted *Lathyrus* seedling height, as the lowest height of 16.44 cm was observed with 30% salinity, while unsalted treatment (0%) significantly produced the greatest seedling height (23.5 cm). Regarding the interaction, the tallest seedlings were obtained by SiNPS-primed seeds under non-stressed condition (25.43 cm). However, the shortest seedlings were given by hydro-primed seed under 30% saline condition.

When it comes to the seedling’s fresh and dry weights, halo-priming application significantly enhanced seedling fresh and dry weights as compared to hydro-priming application. SiNPs-application significantly produced the highest fresh and dry weights (Figure 2). Seawater treatments led to a significant and gradual decrease in fresh and dry weights with increased seawater levels, as the lowest weights were produced by the highest seawater level (30%). In terms of the interaction, the SiNPS-primed seeds under non-stressed condition significantly produced the heaviest fresh (0.47 g) and dry (0.043 g) weights. However, the lowest fresh (0.17 g) and dry (0.018 g) weights were obtained by hydro-primed seed under 30% saline condition.

### 2.3. Leaf Water Status 

**Figure 3:**
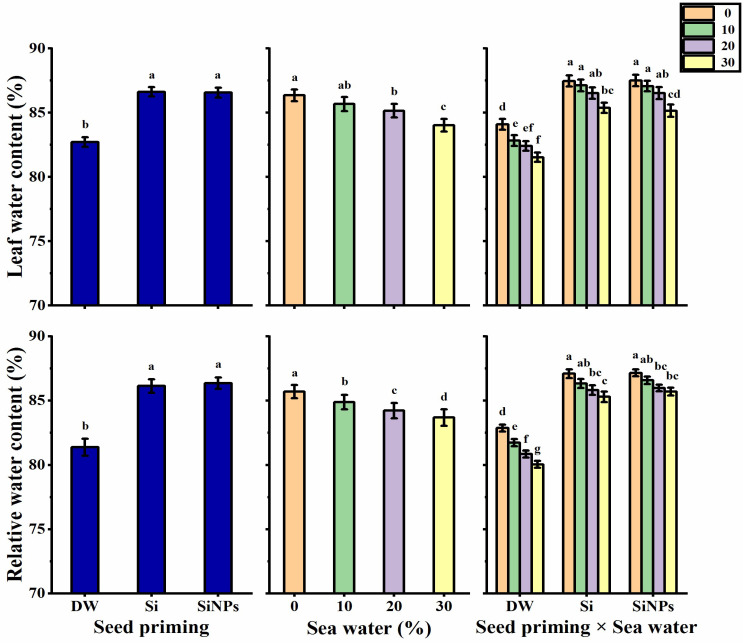
Effect of seed priming on leaf water content (%) and relative water content (%) of *Lathyrus odoratus* leaves irrigated with seawater levels (0, 10, 20, and 30%). Data are mean value ± SE. Bars with different letters are significantly different at *p* ≤ 0.05 level.

The water status of *Lathyrus* leaves (leaf water content; LWC) and relative water content (RWC) was significantly enhanced by halo-priming application as compared with the hydro-priming application (Figure 3). Concerning seawater treatments, increasing seawater levels led to a significant and gradual decrease in the LWC and RWC values. Leaf water status was higher with SiNPS-primed seeds under non-stressed condition, which recorded 87.51 and 87.16% for LWC and RWC, respectively. Meanwhile, a lower leaf water status was reported with hydro-primed seeds under 30% saline condition, with respective LWC and RWC of 81.53 and 80.05%.

### 2.4. Photosynthetic Pigments

**Figure 4:**
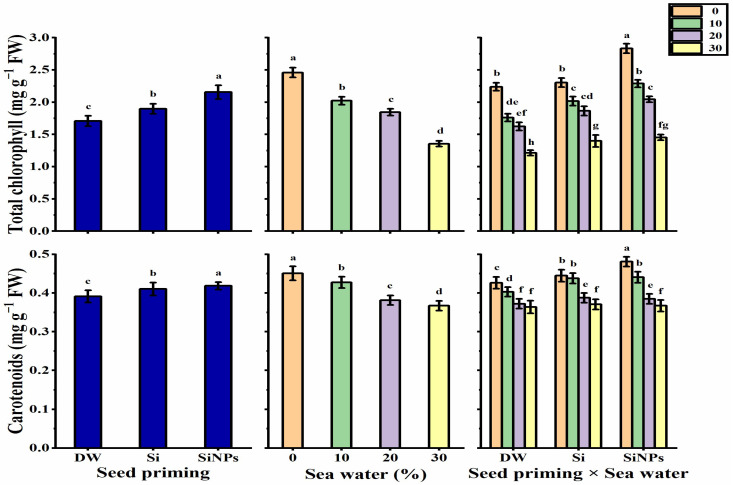
Effect of seed priming on total chlorophyll (mg g^−1^ FW) and carotenoids (mg g^−1^ FW) contents of *Lathyrus odoratus* leaves irrigated with seawater levels (0, 10, 20, and 30%). Data are mean value ± SE. Bars with different letters are significantly different at *p* ≤ 0.05 level.

Leaf photosynthetic pigments (total chlorophyll and carotenoids) were significantly impacted by seed priming and seawater treatments (Figure 4). In this regard, SiNPs produced the highest chlorophyll and carotenoids levels, while the lowest pigment concentrations were obtained by hydro-priming application. Leaf photosynthetic pigments were inversely proportional to seawater treatments, since total chlorophyll and carotenoids content decreased significantly as seawater level increased, reaching its lowest values under 30% salinity (1.36 and 0.367 mg g^−1^ FW for total chlorophyll and carotenoids, respectively). The treatment of SiNPS-primed seeds under non-stressed conditions significantly produced the highest chlorophyll (2.834 mg g^−1^ FW) and carotenoids (0.481 mg g^−1^ FW) concentrations, with the least chlorophyll (1.214 mg g^−1^ FW) and carotenoids (0.364 mg g^−1^ FW) concentrations observed with the treatment of hydro-primed seeds under 30% saline conditions.

### 2.5. Biochemical Parameters

#### 2.5.1. Total Carbohydrates

**Figure 5:**
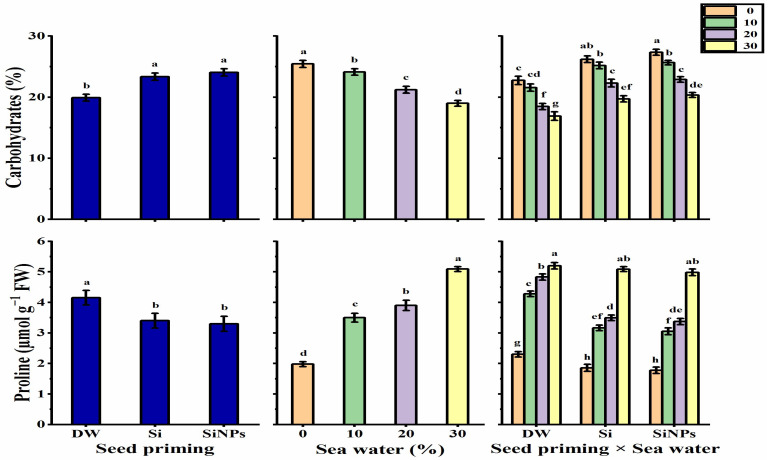
Effect of seed priming on carbohydrates (%) and proline (μmol g^−1^ FW) contents of *Lathyrus odoratus* leaves irrigated with seawater levels (0, 10, 20, and 30%). Data are mean value ± SE. Bars with different letters are significantly different at *p* ≤ 0.05 level.

Seed priming application significantly enhanced total carbohydrate content in *Lathyrus* leaves (Figure 5). Halo-priming application with Si or SiNPs significantly produced higher carbohydrates than hydro-priming application, although there was a non-significant difference between both materials (Si or SiNPs). Total carbohydrates decreased gradually as seawater level increased, reaching its lowest record under 30% salinity, which was roughly 25.35% less than unsalted seedlings (0%). The treatment of SiNPS-primed seeds under non-stressed conditions significantly presented the highest carbohydrate value (27.36%), while the least carbohydrate value was observed with hydro-primed seeds under 30% saline conditions (16.91%).

#### 2.5.2. Proline Content

The results illustrated in Figure 5 show the effect of different seed priming applications on the proline content produced in *Lathyrus* leaves subjected to seawater treatments. Proline content has been decreased by the applications of halo-priming, as the lowest value was noticed with SiNPs-application (3.29 μmol g^−1^ FW), while it was increased by 20.6% when a hydro-priming application was used. Seawater levels caused remarkable variations in the proline values. A significant and gradual increase was observed with increasing seawater levels, reaching the highest proline content under 30% salinity (5.09 μmol g^−1^ FW) against the lowest proline content found in unsalted *Lathyrus* leaves (1.98 μmol g^−1^ FW). The maximum proline content was recorded with the treatment hydro-primed seeds under 30% salinity (5.20 μmol g^−1^ FW), while the lowest proline content was given by SiNPS-primed seeds under non-stressed conditions (1.78 μmol g^−1^ FW).

#### 2.5.3. Total Phenols and Flavonoids Contents

**Figure 6:**
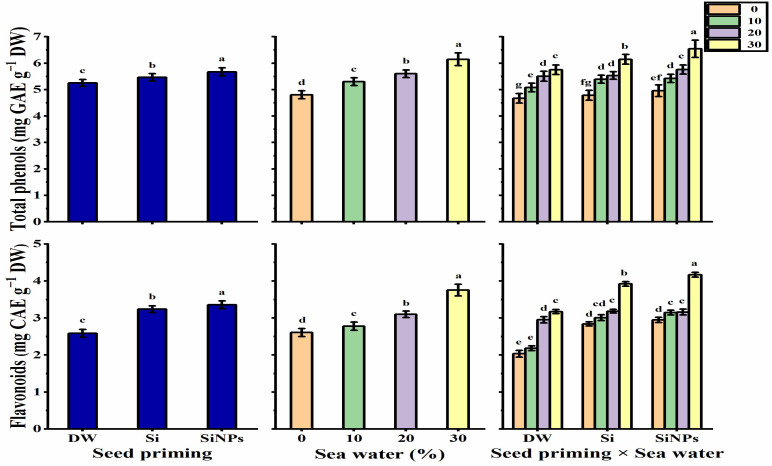
Effect of seed priming on total phenols (mg GAE g^−1^ DW) and flavonoids (mg CAE g^−1^ DW) contents of *Lathyrus odoratus* leaves irrigated with seawater levels (0, 10, 20, and 30%). Data are mean value ± SE. Bars with different letters are significantly different at *p* ≤ 0.05 level.

The total phenols and flavonoids content significantly increased after halo-priming applications (with Si or SiNPs) as compared with hydro-priming applications (Figure 6). SiNPs significantly recorded the highest levels in this respect. Increasing seawater levels caused a significant and growing increase, reaching the highest values when plants were irrigated with 30% seawater. The highest levels of phenols (6.544 mg GAE g^−1^ DW) and flavonoids (4.170 mg CAE g^−1^ DW) were detected in *Lathyrus* leaves that had been treated with SiNPS-primed seeds under 30% salinity. Meanwhile, the hydro-primed seeds under non-stressed conditions identified the lowest values in this regard (4.670 mg GAE g^−1^ DW and 2.035 mg CAE g^−1^ DW for phenols and flavonoids, respectively).

#### 2.5.4. Oxidative Damage Induced 

**Figure 7:**
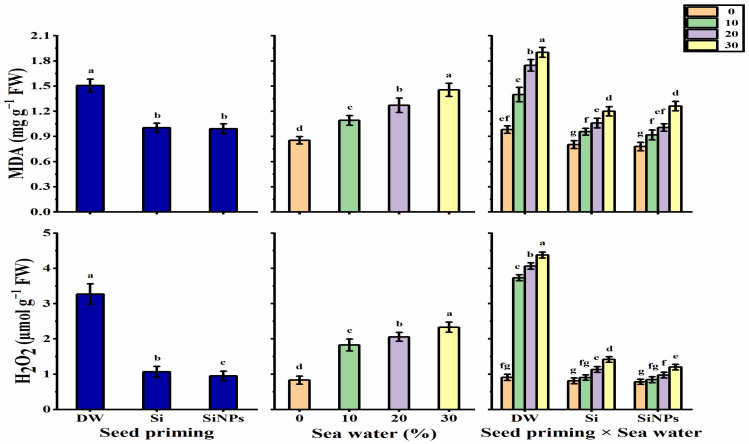
Effect of seed priming on MDA (mg g^−1^ FW) and H_2_O_2_ (μmol g^−1^ FW) contents of *Lathyrus odoratus* leaves irrigated with seawater levels (0, 10, 20, and 30%). Data are mean value ± SE. Bars with different letters are significantly different at *p* ≤ 0.05 level.

Lipid peroxidation (MDA) and H_2_O_2_ content decreased significantly more with halo-priming applications than with hydro-priming applications (Figure 7), as SiNPs-application significantly produced the lowest values (34.2% for MDA and 70.9% for H_2_O_2_ lower as compared to hydro-priming applications) in this respect. The MDA and H_2_O_2_ values gradually increased with increasing seawater levels, with the maximum values being obtained under 30% seawater. The lowest levels of MDA and H_2_O_2_ were presented by SiNPS-primed seeds under non-stressed conditions, but hydro-primed seeds under 30% saline conditions significantly caused the maximum values in this respect.

#### 2.5.5. Antioxidant Enzyme Activities

**Figure 8:**
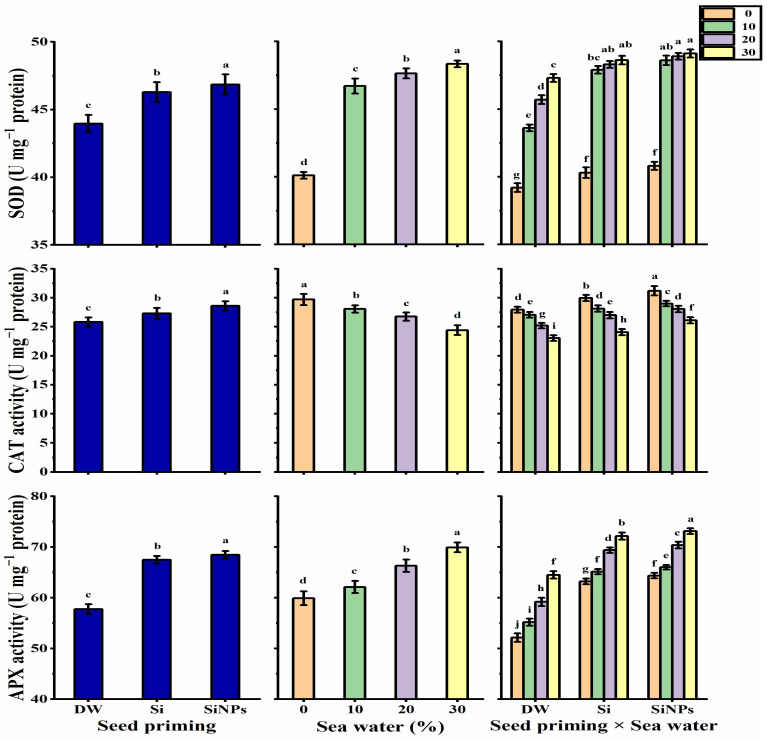
Effect of seed priming on SOD (U mg^−1^ protein), CAT (U mg^−1^ protein), and APX (U mg^−1^ protein) activities of *Lathyrus odoratus* leaves irrigated with seawater levels (0, 10, 20, and 30%). Data are mean value ± SE. Bars with different letters are significantly different at *p* ≤ 0.05 level.

Applications of halo-priming significantly increased the activities of superoxide dismutase (SOD), catalase (CAT), and ascorbate peroxidase (APX) enzymes in *Lathyrus* leaves relative to hydro-priming applications, as the highest values were produced by the SiNPs-application (Figure 8). The maximum levels of SOD and APX activities were observed by 30% salinity, while CAT activity decreased with increasing seawater levels. In terms of the interaction effect, the highest levels of SOD and APX were detected by SiNPS-primed seeds under 30% salinity, while the highest CAT value was given by the treatment of SiNPs-primed seeds under non-stressed conditions. On the other hand, the lowest SOD and APX activities were obtained by hydro-primed seeds under non-stressed conditions and by hydro-primed seeds under 30% saline conditions for CAT activity.

### 2.6. Ion Contents

**Figure 9:**
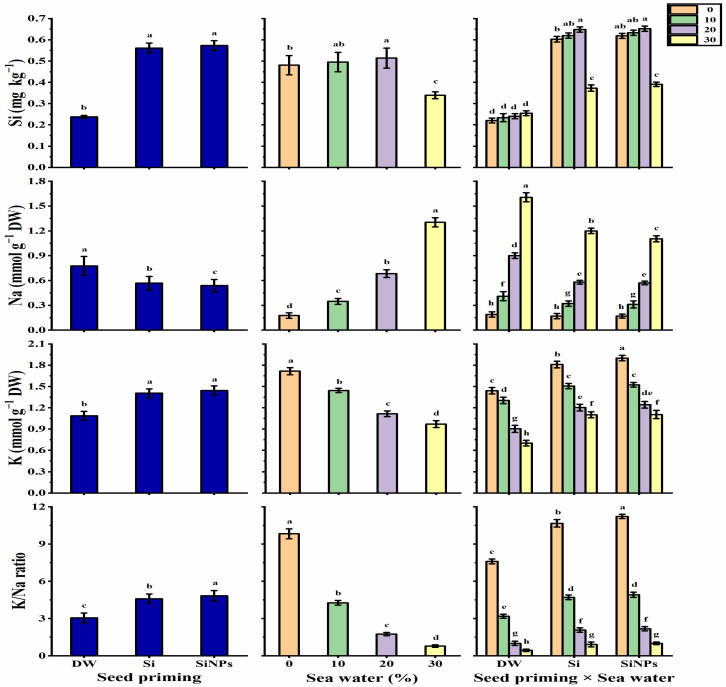
Effect of seed priming on Si (mg Kg^−1^), Na (mmol g^−1^ DW), K (mmol g^−1^ DW) contents, and K/Na ratio (g) of *Lathyrus odoratus* leaves irrigated with seawater levels (0, 10, 20, and 30%). Data are mean value ± SE. Bars with different letters are significantly different at *p* ≤ 0.05 level.

The results depicted in Figure 9 indicate the content of Si, K^+^, and Na^+^ accumulated in *Lathyrus* leaves, and the K^+^/Na^+^ ratio in response to priming applications under seawater treatments. Leaf Si content was significantly elevated following halo-priming applications, as compared with the hydro-priming application. Increasing seawater levels caused an increase in Si content reaching its greatest value at 20% salinity, and then decreased after that. Regarding the interaction effect, both Si and SiNPS-primed seeds under 20% saline conditions significantly showed the highest values of Si in *Lathyrus* leaves. The hydro-priming application significantly exhibited the highest Na^+^ content, but SiNPs-application significantly recorded the lowest Na^+^ value (30.7% less than hydro-priming application). A gradual and significant increase was noticed with increasing salinity levels. Concerning the interaction, the maximum Na^+^ content was obtained by hydro-primed seeds under 30% salinity. Meanwhile, the Si and SiNPS-primed seeds under non-stressed conditions had the lowest Na^+^ content.

**Figure 10:**
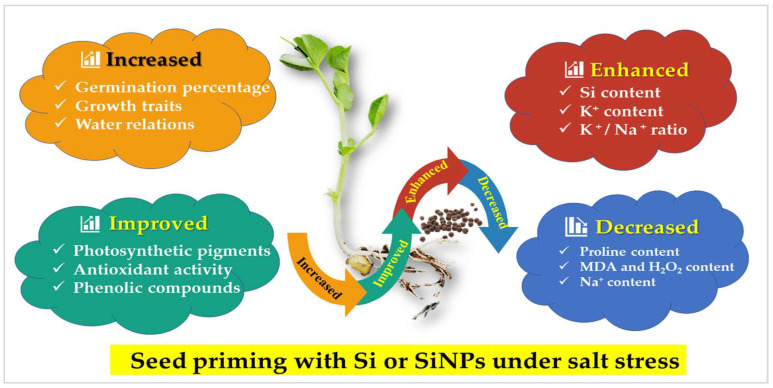
The mechanisms involved in seed primed *Lathyrus* (with Si or SiNPs) for gaining salt stress tolerance.

The K^+^ content and K^+^/Na^+^ ratio significantly increased with halo-priming applications as compared with hydro-priming applications, which significantly recorded the lowest K^+^ value and K^+^/Na^+^ ratio. A gradual and significant decrease was observed with increasing seawater concentration. The treatment of SiNPS-primed seeds under non-stressed conditions significantly exhibited the highest K^+^ value (1.9 mmol g^−1^ DW) and K^+^/Na^+^ ratio (11.23). On the other hand, the treatment of hydro-primed seeds under 30% saline conditions significantly exhibited the lowest K^+^ (0.70 mmol g^−1^ DW) and K^+^/Na^+^ ratio (0.44). The mechanisms involved in seed priming with Si or SiNPs for gaining salt stress tolerance in *Lathyrus* seedlings are presented in Figure 10.

## 3. Discussion 

Plant growth and development, as well as productivity, are predicted by seed germination. In the current research study, salt stress decreased GP and prolonged MGT, as well as causing a pronounced inhibition of *Lathyrus* seedling growth. Salinity stress decreased seed germination as a result of limited water absorption, slowed the breakdown of seed storage material, and inhibited the production of storage proteins [26,27]. Basically, salinity decreases cell expansion and division, and inhibits physiological and biochemical processes [28,29], which causes a reduction in the photosynthetic rate, dry matter accumulation, and total carbohydrates content. Furthermore, salt stress reduces chlorophyll synthesis and activates degraded chlorophyllase enzymes [30,31]. In accordance with our results, the negative effects of salt on plant growth features have been previously reported by Al-Yasi et al.; Attia et al., [32,33].

In contrast, Si and SiNPs treatments improved the seed germination of *Lathyrus* seeds. Si was found to be directly linked to the physiological process of seed germination in *Glycyrrhiza uralensis* under saline conditions [29]. Halo-priming with SiNPs exhibited the highest MGT value. Similar results were obtained by Siddiqui and Al-Whaibi [34].

The increase in mean germination time following SiNPs seed priming may be due to the ability of silica to absorb moisture from the surroundings [35]. Furthermore, 1 g of SiO_2_ nanoparticles (7 nm) has a surface adsorption of 400 m^2^ [36]. Increasing the seawater levels caused a reduction in the GP and an increase in the MGT. Salts negatively impact the levels of endogenous phytohormones, which essentially inhibit seed germination and plant growth [37].

In our study, halo-priming applications enhanced plant height, shoot FW and DW, leaf water status, leaf pigments, and total carbohydrate under salinity stress, more than the hydro-priming application. Silicon promotes cell elongation and division, leading to elevated plant height [38,39].

Under salinity conditions, Si enhances photosynthetic activity by decreasing ion toxicity and ROS content, preserving the structure and function of the organelles responsible for photosynthesis [37], maintaining stomatal conductance, transpiration, membrane permeability, net photosynthesis, and chlorophyll levels [29]. Moreover, Si decreases the leaf curve angle and increases leaf flatness, allowing for increasing light interception and more photosynthetic pigments [39], and thus more carbohydrates and dry matter accumulation. Silicon enhances growth performance directly, through blocking Na^+^ transport, and indirectly, through different physiological processes that alleviate the negative effects of salinity [37]. Increased biomass production is the main indicator of plant resistance [40].

Leaf water status is a good indicator of water relations in plants. Preserving a good water status in plant cells helps to maintain osmotic adjustments and the activity of metabolic processes, and increases plant resistance under salinity stress [41,42]. Under seawater treatments, the LWC and RWC of *Lathyrus* leaves decreased gradually with increasing seawater levels. Salts decrease the osmotic pressure in plant cells, so they have a negative effect on water uptake by plant roots [43]. Halo-priming treatments exhibited better leaf water status. Silicon decreases plant transpiration [29] due to Si accumulation as external layers above cell walls of leaves and stems, leading to thicker leaves and stem cuticle. In addition, Si improves stem hydraulic conductance [39]. Silicon alters the osmotic pressure, which increases plant tolerance under salinity stress conditions [44,45]. Higher water content in Si-plants grown under saline conditions is mainly associated with salt dilution inside the plant, leading to plant growth improvements [44]. Therefore, it can be concluded that Si improves the leaf water status and mitigates the osmotic stress induced by seawater treatments in *Lathyrus* leaves.

Under seawater treatments, proline content gradually increased with increased seawater levels. Proline is normally produced in high amounts under salinity stress [46]. Proline plays a vital role in osmotic adjustment, sub-cellular structure protection, enzyme activities, and can also increase the cellular turgor pressure, which is responsible for cell expansion under salinity conditions [47,48]. Halo-priming treatments presented lower levels of proline. Si decreases proline accumulation under salinity stress, which indicates the role of Si in alleviating damage caused by salts [49]. Total phenols and flavonoids contents of the metabolic *Lathyrus* leaf extract are gradually increased with higher seawater concentrations, because salts stimulate phenolic compound synthesis.

Phenolic compounds, including flavonoids, are among the many sources of antioxidants in plants and are considered to be a response to protect against the oxidative damage caused by salts [50]. The chemical structure of phenols enables them to deactivate singlet oxygen and act as hydrogen donors, allowing them to scavenge ROS [51,52]. Halo-priming treatments demonstrated a significant increase in the total phenolic compound, and SiNPs were superior in this respect which may be due to the nanoscale size of the insoluble SiNPs accumulated in the epidermis. This may allow constitutional phenols to be produced on the large adsorption surfaces of epidermal cells [53,54].

Plants alleviate the oxidative damage that occurs under saline conditions through nonenzymatic (phenolic compounds) generation [55]. These processes play a vital role in protecting plant cells from the oxidative damage [56] that occurs at the cell membrane [28,57], and in ion balance and water status [58].

In our study, plants subjected to salt stress exhibited higher H_2_O_2_ and MDA levels; however, halo-priming treatments revealed a significant decrease in this regard. H_2_O_2_ negatively impacts cell membrane lipids and causes oxidative damage, which was evidenced in the increased MDA accumulation (the indicator of lipid peroxidation) [59]. Si significantly increased antioxidant enzyme activities (CAT, APX, and SOD) and reduced ROS accumulation (H_2_O_2_) in *Lathyrus* leaves. In the case of saline conditions, the overproduction of reactive oxygen species (ROS) exposes plant cells at risk by inducing lipid peroxidation, protein oxidation, nucleic acid damage, enzyme inhibition, and the initiation of the programmed cell death process [56]. The antioxidant enzymes CAT, SOD, and APX, as well as non-enzymatic antioxidant substances (phenolic and flavonoids compounds, proline, and carotenoids), had the ability to scavenge ROS compounds [55,56]. Si mitigates the negative effects of salt stress by enforcing the antioxidant defense capability, which reduces lipid peroxidation and plasma membrane permeability [49].

Silicon increases plant tolerance by regulating stress-related phytohormone biosynthesis [60]. Using seawater to irrigate *Lathyrus* seedlings leads to increasing Si and Na^+^ in the leaves and reduces the amount of K^+^, and the K^+^/Na^+^ ratio. Elevated K^+^/Na^+^ ratio stimulates plant tolerance to salinity stress [61]. Under salinity stress, the K^+^/Na^+^ ratio decreased due to Na^+^ toxicity which inhibits K^+^ uptake. This was also caused by Na^+^ and K^+^ competition on binding sites [62]. Halo-priming treatments showed less Na^+^ and more K^+^ accumulation in *Lathyrus* leaves than hydro-priming applications. Silicon increased K^+^ concentration in plant cells under saline conditions [49]. Under salinity stress, Si reduces the net rate of Na^+^ uptake and accumulation in plants [63]. The deposited Si beneath the cell walls of the roots, binds with Na^+^, causing an increase in K^+^ uptake and a reduction in Na^+^ transported to the plant shoots [49]. Application of Si substantially increased K^+^ and decreased Na^+^ content in the cytoplasm because of the activity of H+-ATPase in the tonoplasts and plasma membrane, as well as H+-PPase activation in tonoplasts under salt-stress conditions [64]. The effects of Si on Na^+^ transport resulted from the blockage of the apoplastic pathway [65], which alleviated Na^+^ toxicity under salt stress.

## 4. Materials and Methods

### 4.1. Location and Plant Materials

This pot study was undertaken at a greenhouse at the Faculty of Agriculture farm, Tanta University, Tanta, Egypt, (latitude of 30°47′ N: and longitude 31°0′ E), during the 2019 and 2020 winter seasons. Mature and uniform *Lathyrus* seeds were surface-sterilized for 5 min using sodium hypochlorite (10%), and then washed with distilled water. The sterilized seeds were divided into three groups for priming with varying solutions; the first group was hydo-primed with distilled water; the second group was halo-primed with sodium silicate (Si) solution at 50 mg/L; and the third group was halo-primed with 20 mg/L of silicon nanoparticle (SiNPs) solution. The seeds were primed for 9 h, and then the primed seeds were naturally air-dried. Each seed group was divided into four groups, the first seed group was irrigated with tap water (1.52 dS m^−1^); the second, third, and fourth groups were subjected to saltinity stress using seawater levels of 10% seawater + 90% tap water (9.33 dS m^−1^), 20% seawater + 80% tap water (14.87 dS m^−1^), and 30% seawater + 70% tap water (21.60 dS m^−1^), respectively. Primed seeds were sown in plastic pots of 25 cm diameter containing 9 kg soil, (10 seeds/pot) on September 15th of both seasons. After sowing, the pots were irrigated three times for the week, in order to reach the saturation percentage with seawater levels of 0, 5, 10, and 15% for the second, third, and fourth groups, respectively, to avoid osmotic shock. From the second week, seawater levels increased to the planned levels of 0, 10, 20, and 30% seawater for the second, third, and fourth group respectively; all pots were irrigated every two days with the seawater levels to reach the field capacity. Some physical and chemical properties of the soil were determined as follows: sand, 67.24%; silt, 11.14%; and clay, 21.62%; EC, 1.66 dS m^−1^; pH, 7.34; Ca^2^^+^, 8.45 meq L^−1^; total N^+^, 0.26%; PO_4_^3−^, 0.041%, and K^+^, 0.06%. Data of growth performance and biochemical analysis were estimated 45 days after sowing.

### 4.2. Seawater Source and Chemicals

Seawater was collected from Marsa Matrouh beach, Mediterranean Sea, Egypt. The water analysis is presented in Table 1. Sodium silicate (Na_2_SiO_3_, 99% purity) was bought from Sigma-Aldrich Chemie (St. Louis, MO, USA), and silicon nanoparticles (15–45 nm, 99.5% purity) were bought from Nano-technology Laboratory, Faculty of Science, Tanta University, Tanta, Egypt).

### 4.3. Experiment Layout

The current study was performed in a factorial randomized complete design. Primed solutions were the first factor, while seawater concentrations were the second factor. The experiment consisted of 12 treatments, with six replicates for each treatment; each replicate consisted of three pots, and the pot contained 10 seeds.

### 4.4. Germination and Growth Characteristics

In order to calculate germinated seeds, the seeds were checked and counted on a daily basis. Seeds were considered germinated when cotyledon appeared above the soil surface. Germination percentage (GP) was estimated on day 18 and calculated according to the following equation:

GP (%) = (number of germinated seeds on day 18/total seeds number) × 100.

Germination speed is expressed as mean germination time (MGT) according to the equation of Ellis and Roberts [66].

MGT = ∑ Dn/∑ n.

where, n refers to the number of germinated seeds on day D0, and D refers to the number of days from the beginning of the germination experiment. The seedlings were left to grow for 27 days, and order to plant height, shoot fresh weight (FW), shoot dry weight (DW) were determined.

### 4.5. Leaf Water Status

To determine the leaf water status, the relative water content (RWC) and leaf water content (LWC) were estimated according to Clarke and Mccaig [67] and Barrs [68] as follows: two leaf samples were separated, and immediately weighed, and the fresh weight (FW) was recorded. After that, the same leaf samples were saturated in distilled water at 4 °C for 24 h and their turgid weight was recorded (TW). Then, the same leaf samples were oven-dried at 70 °C for 48 h until reaching a constant and the dry weight (DW) was recorded. RWC and LWC were determined using the following formulae:

LWC = ((FW − DW) ÷ FW) × 100

RWC = (FW − DW) ÷ (TW − DW) × 100

### 4.6. Photosynthetic Pigments

Leaf pigments of sweet pea were estimated using methanol, as previously described by El-Serafy [69] according to Dere, et al. [70]’s protocol. Samples of fresh leaves (0.2 g) were homogenized in 96% methanol (10 mL) for 1 min. The homogenate was filtrated, and centrifuged for 10 min at 2500 rpm. The supernatant was used for chlorophyll determination using an UVVIS spectrophotometer at wavelengths of 666 nm, 653 nm, and 470 nm for chlorophyll a, b, and total carotenoids, respectively, and its contents are presented in mg g^−1^ FW.

### 4.7. Biochemical Parameters

#### 4.7.1. Total Carbohydrates

Total carbohydrates were estimated as described by Weinmann [71]. In brief, 0.5 g of dried leaves was mixed with 1 N sulfuric acid (10 mL) in a glass tube. The tube was bolted and heated at 100 °C in an oven over night. The total carbohydrates content was estimated colorimeterically following the method of Dubois, et al. [72]. A total of 1 mL of sugar solution was added to phenol solution 5% (1 mL) followed by 5.0 mL sulfuric acid. The mixture was shaken thoroughly and maintained at 23–30 °C in a water bath for 20 min. The developed color was determined at 490 nm wavelength throughout the UVVIS spectrophotometer analysis, and total carbohydrates content is expressed as percentage.

#### 4.7.2. Proline Content

The proline content was determined as explained by Bates et al. [73] protocol. Briefly, proline extract, ninhydrin acid and glacial acetic acid at volumes of 2, 2, 2 mL were mixed and incubated in a boiling water bath for 1 h, and then were placed in an ice bath. The 520 nm wavelength was used for absorbance determination. A standard curve was constructed at certain levels of authentic proline.

#### 4.7.3. Phenols and Flavonoids Determination

Total phenol content in leaves was determined with the Folin–Ciocalteu procedure using the standard of gallic acid, as previously described by El-Serafy and El-Sheshtawy [74], according to Boateng, et al. [75], with some modifications. Dried leaf samples (1 g) were mixed with 50 mL of methanol 80% and macerated at room temperature for two days. The extract was maintained below 4 °C for total phenol estimation after being fully solvent removed. A total of 1 mL of leaf extract was mixed with Folin–Ciocalteau reagent at a volume of 1 mL, and left to stand for incubation (5 min). Then, a 2 mL of Na_2_CO_3_ solution (70 g/L) was supplemented. Again, it was left for incubation at 25 °C for 2 h. Thereafter, the absorbance was estimated at a wavelength of 750 nm. Phenolic content is expressed as mg GAE g^−1^ DW. The methods of Boateng et al. [75] and Talukdar [76] were used for total flavonoids estimation based on the aluminum chloride procedure. The mixture of 0.5 mL of the extract and 0.5 mL of aluminum chloride (2%) was left for incubation at room temperature for 45 min. Then, the absorbance was determined at 420 nm wavelength for the resulting mixture. Catechin (CAE) was used to calculate the standard curve, and the flavonoids content is expressed in mg CAE g^−1^ DW.

#### 4.7.4. Lipid Peroxidation Estimation 

The content of MDA was determined and utilized as an indicator of lipid peroxidation in *Lathyrus* leaves under tested treatments. MDA was estimated as described by Heath [77] with little modification. A sample of 0.5 g of fresh leaves was centrifuged for 10 min at 12,000× *g* after mixing with 5.0 mL of TCA 5% (*w*/*v*). A total of 2 mL of the extract was added to 2 mL of TBA (0.6%), and then heated for 10 min in a water bath (95 °C). The absorbance was estimated at 532 and 600 nm wavelengths. MDA content (mg g^−1^ FW) was calculated using the following formula

MDA content = 6.45 × (A532 − A600) − 0.56 × A450.

#### 4.7.5. H_2_O_2_ Content

The content of H_2_O_2_ in *Lathyrus* leaves was estimated as described by Patterson [78]. A total of 0.5 g of each sample was homogenized in 6 mL of chilled acetone (100%) and centrifuged for 10 min at 4 °C, at 12, 000 g. Then, 1 mL of the final extract was added to 5% Ti (SO_4_)_2_ (0.1 mL), and 0.2 mL of NH_4_OH solution. Thereafter, the mixture was centrifuged for 10 min at 3000 *g*. A total of 4 mL of H_2_SO_4_ (2 M) was used for dissolving. The optical density was measured at 412 nm wavelength. For calibration, a standard curve was formulated using various levels of H_2_O_2_, and the obtained data were recorded as μmol g^−1^ FW.

#### 4.7.6. Antioxidant Enzyme Activities

Fresh leaves were used for enzyme extraction as described by Murkherje and Choudhuri [79], with simple modifications. A total of 0.3 g of fresh leaves was ground with 0.1 mM potassium phosphate buffer (PBS) solution (pH 7.8) and made into a homogenate under ice conditions, which was centrifuged at 10,000 *g* for 20 min at 4 °C. The obtained supernatant was retained at 4 °C for enzyme activity determination.

For superoxide dismutase (SOD, EC 1.15.1.1) determination, the nitro blue tetrazolium procedure of Giannopolitis and Ries [80] was utilized as follows: 0.1 mL of enzyme extract was mixed with 100 mM PBS (pH 7.8), Na_2_CO_3_ 1.5 mM, NBT 2.25 mM, methionine 200 mM, EDTA 3 mM, riboflavin 0.06 mM, in addition to distilled water. The reactions tubes with or without enzymes extract (control) were illuminated for 10 min with a 15 W fluorescent lamp; the blank tubes were not illuminated. The absorbance was spectrophotometrically estimated at the 560 nm wavelength. SOD unit was expressed as the amount of enzyme required to inhibit the rate of NBT reduction by 50% in the controls tubes.

Catalase (CAT, EC 1.11.1.6) activity determination was run according to Aebi [81]. In brief, 3 mL of the reaction solution was mixed with 50 mmol L^−1^ PBS (pH 7.0) and 10 mmol L^−1^ of H_2_O_2_ solution. Then, the enzyme activity was estimated by calculating the amount of H_2_O_2_ which was consumed at 240 nm for 2 min.

Ascorbate peroxidase (APX, EC 1.11.1.11) determination was conducted as described by Nakano and Asada [82]. A fresh leaf sample (0.1 g) was mixed with 0.2 mL of extraction buffer, which consisted of 3.0 mM EDTA and 0.1 M Na-phosphate adjusted to pH 7.0, and mixed with 1.0% Triton X-100 and 1.0% polyvinylpyrrolidone. Thereafter, the mixture was centrifuged at 10,000 *g* for 20 min. The absorbance estimation was determined at 290 nm wavelength. The reaction buffer consisted of 0.1 mM H_2_O_2_, 0.5 mM ascorbate, 0.05 mL of extract containing enzyme, and 0.1 mM EDTA mL^−1^; the reaction was performed at 25 °C for 5 min. The coefficient of absorbance of 2.8 mM^−1^ cm^−1^ was used to calculate the activity of APX.

#### 4.7.7. Ion Estimation

Si was estimated according to Snyder [83] using ICP-OES. Briefly, 0.1 g of ground leaf was mixed with 3.0 mL of NaOH (18.5 M) in 55 mL TeflonH vessels. Then, the mixture was heated up to 200 °C for 15 min in a microwave, and maintained at this temperature for 15 min. A total amount of 2 mL H_2_O_2_ was added to the mixture after cooling to the room temperature. Then, the mixture was re-heated to 200 °C for 15 min, and left for 5 min at 200 °C. The mixture was filtered after cooling. A total of 9 mL of deionized water was added to 1 mL of the filtrate and injected into the ICP-OES for determination. Dried leaf samples (0.5 g) were digested with 0.5% HNO_3_ according to Deal [84]. Sodium (Na^+^) and potassium (K^+^) contents were determined using flame photometry, and are expressed as mmol g^−1^ DW.

### 4.8. Statistical Analysis

The present investigation was designed in a complete randomized layout in factorial arrangement with two factors. The data sets of both tested seasons were collected and subjected to ANOVA using the SPSS program Base 9, SPSS Inc. USA. A combined analysis was performed. Duncan multiple rang test was used at *p* ≤ 0.05 probability level to compare mean differences according to Waller and Duncan [85]. The results were presented as means ± SE.

## 5. Conclusions

This research study investigated the potential of seed priming with Si and SiNPs to enhance salt stress tolerance in *Lathyrus*. Halo-priming application with SiNPs effectively exhibited improved salt tolerance against seawater treatments in *Lathyrus* seedlings. Despite SiNPs application increasing MGT, their seedlings showed similar characteristics to the seeds primed with Si, in terms of growth characteristics and salt stress tolerance. Halo-priming with SiNPs improved growth traits, carbohydrates accumulation, photosynthetic pigments content, K^+^/Na^+^ ratio, and enzymatic (SOD, APX and CAT) and nonenzymatic (phenolic compounds) generation in salted seedlings. The decrease in MDA and H_2_O_2_ contents in halo-priming treatments protected the cell membrane from deterioration. Using the seed priming technique for salt stress tolerance in *Lathyrus* seeds not only significantly influenced plant growth and resistance, but also enhanced their aesthetic value and ornamental appearance. This is because the seed priming technique helped maintain the dry matter and carbohydrate accumulation values, and preserved the physiological processes under seawater treatments.

## Figures and Tables

**Table 1:** A chemical analysis of seawater.

Component	EC (dS/m)	pH	HCO_3_ (meq/L)	CL (meq/L)	SO_4_^−2^ (meq/L)	Ca^+2^ (meq/L)	Mg^+2^ (meq/L)	Na^+^ (meq/L)	K^+^ (meq/L)
**Concentration**	40.51	6.81	5.59	415	74.35	42.10	14.57	435.35	1.34
